# Review: Techniques in Egocentric Multi-View Image Analysis: Advances, Challenges, and Future Directions

**DOI:** 10.3390/jimaging12070324

**Published:** 2026-07-17

**Authors:** Duc Tri Phan, Hong Duc Nguyen

**Affiliations:** 1 Institute of Research and Development, Duy Tan University, 254 Nguyen Van Linh, Da Nang 550000, Vietnam; phanductri1@duytan.edu.vn; 2 School of Electrical and Electronic Engineering, Nanyang Technological University, Singapore 639798, Singapore

**Keywords:** egocentric multi-view, cross-view feature fusion, multi-view stereo, human–object interaction, novel view synthesis, open-world detection, 3D hand/object tracking, egocentric action recognition

## Abstract

Egocentric multi-view image analysis refers to the processing of utilizing synchronized video streams captured from multiple wearable cameras worn on the head or body, providing complementary first-person perspectives of dynamic, real-world interactions. Unlike single-view egocentric vision, which may suffer from severe occlusions, motion blur, and limited field-of-view or traditional fixed-camera multi-view setups (assuming static geometry and controlled environments), egocentric multi-view systems leverage body-worn rigs to enable a more robust and flexible 3D understanding in open-world, mobile scenarios. In this work, we present a systematic survey of advancements in cross-view feature fusion, geometric consistency enforcement, open-world detection, human–object interaction (HOI) modeling, action segmentation, 3D reconstruction, and novel-view synthesis specifically tailored to wearable multi-camera platforms. Key datasets released between 2024 and 2026—including HOT3D (833 min of synchronized multi-view hand/object interactions from Project Aria and Quest 3), MultiEgo (first multi-egocentric dataset for 4D social scene reconstruction), and Ego-1K (large-scale 12-camera rig for dynamic 3D video synthesis) are thoroughly examined alongside an analysis of integrations with large language models (LLMs) and vision–language models that drive performance gains, typically in the 15–30% range over single-view baselines in hand tracking, HOI recognition, and reconstruction fidelity, although we show through a consolidated meta-analysis that this gain is task-dependent: larger for geometry-bottlenecked tasks such as in-hand object lifting, and smaller, method-dependent, or occasionally negative for semantic-recognition tasks such as keystep recognition under naive view fusion. These methods cover work in multi-view stereo, cross-view learning, and novel-view synthesis while addressing several real-time wearable constraints. Practical applications such as immersive Augmented Reality/Virtual Reality (AR/VR), assistive robotics, and healthcare monitoring are also discussed together with the challenges in motion calibration, benchmark diversity, and edge deployment ability. Thus, in this review, we attempt to fill a critical gap by focusing exclusively on wearable multi-view systems in an open-world setting, synthesizing the latest literature to chart future directions toward more embodied and continual learning agents.

## 1. Introduction

### 1.1. The Growth of Egocentric Vision

Egocentric vision, also known as first-person vision, refers to capturing and analyzing visual data from a wearable camera mounted on the human body—typically on the head or chest [1]. This setup provides a direct view of the wearer’s interactions with the surrounding environment. Unlike traditional third-person (allocentric) computer vision, which relies on fixed or externally mounted cameras that observe scenes from a detached viewpoint, egocentric vision records dynamic scenes from the user’s own perspective, closely resembling natural human visual experience [2]. In addition, the rapid advancement in technology for these wearable devices—such as Augmented Reality/Virtual Reality (AR/VR) headsets, smart glasses, and multi-camera rigs—has accelerated progress in this field [3]. Furthermore, the formulations of large-scale datasets have played a crucial role in this growth. For example, Ego4D [4] provides 3670 h of daily-life video collected from 923 participants across 74 locations worldwide, greatly expanding the availability of first-person data and supporting research on embodied Artificial Intelligence (AI). Ego–Exo4D [5] further extends this direction by offering synchronized egocentric and exocentric recordings for skilled activity understanding. In addition, open source datasets such as HOT3D [6] have also been released, containing 833 min of synchronized multi-view hand–object interaction recordings. These resources provide high-quality, multimodal data that enable more robust modeling and evaluation, and they are also supported by hardware miniaturization and multimodal sensor integration. Modern research platforms, such as Meta’s Project Aria glasses [7], integrate high-resolution RGB and monochrome cameras, inertial measurement units (IMUs), gaze tracking, and on-device processing. These features make egocentric data capture practical in unconstrained, real-world environments. Recent surveys [3,8] emphasize that egocentric vision connects computer vision, machine learning, and cognitive science, offering deeper insights into human actions, object manipulation, and environmental interaction compared to static camera systems. The main advantage of egocentric vision is its ability to provide immersive, human-centered perception that closely matches first-person experience. Its key benefits include:

- Reduced occlusion and consistent viewpoints for object manipulation: Hands and manipulated objects usually appear near the center of the frame, making it easier to analyze human–object interactions (HOI), recognize actions, and learn skills. These tasks are often more difficult in third-person views.
- Natural embodiment and contextual awareness: By capturing the wearer’s vision and actions in real time, egocentric systems allow AI models to infer intentions, anticipate actions, and provide context-aware assistance. This is especially important for augmented reality (AR) and virtual reality (VR), where digital content must respond smoothly to gaze direction, hand movement, and environmental changes.
- Support for embodied intelligence and human–AI collaboration: Egocentric data supports applications in robotics (e.g., imitation learning from human demonstrations), assistive technologies, healthcare monitoring, and social behavior analysis. Wearable systems can act as memory aids, provide step-by-step guidance, or improve productivity in training and manufacturing environments.
- Foundation for hybrid and multi-view systems: Although early single-camera setups faced challenges such as motion blur and limited field of view, these limitations are increasingly addressed through wearable multi-camera systems. Such systems enable more reliable 3D reconstruction, novel-view synthesis, and cross-view feature fusion in dynamic, real-world scenarios.

In summary, the rapid growth of egocentric vision—driven by large-scale datasets, advanced wearable hardware, and shared community benchmarks—positions first-person perception as a core technology for next-generation immersive and interactive AI systems. It shifts the focus of computer vision from passive observation to active, embodied understanding, with important implications for AR/VR, robotics, and human-centered computing.

### 1.2. Limitations of Single-View Egocentric Approaches

Despite its rapid growth and unique advantages for immersive interaction, single-view egocentric vision suffers from several inherent limitations that hinder robust scene understanding, particularly in dynamic, open-world environments. One of the most prominent challenges is severe motion blur, which arises from rapid head and body movements typical in first-person activities. Sharp head rotations and forward movement cause the camera to move quickly, resulting in blurred frames that degrade the quality of visual features and complicate tasks such as object recognition, hand tracking, and action segmentation. Studies [9,10] consistently identify motion blur as a key factor that reduces tracking performance in egocentric videos compared to third-person views. Another critical issue is frequent and severe object occlusions. In natural interactions, the wearer’s hands, arms, or manipulated objects often partially or fully obscure the scene, especially during close-range human–object interactions (HOI). Additionally, dynamic viewpoint changes can cause objects or body parts to temporarily disappear from the field of view and reappear later, leading to fragmented and incomplete information. Surveys [11,12] highlight occlusion as the major challenge in egocentric videos, posing substantial interference to the accuracy and stability of models for pose estimation, tracking, and scene understanding. Furthermore, the various positions of viewpoints further exacerbate these problems. The egocentric camera, typically mounted on the head or body, undergoes continuous and unpredictable shifts in perspective due to natural wearer motion. This results in unusual viewpoints, limited field-of-view, extreme scale variations, and rapid foreground–background switching, which introduce significant appearance changes and reduce parallax for reliable 3D reasoning [13]. Such variability makes it difficult to maintain consistent object or hand representations across frames and limits the effectiveness of monocular depth estimation or SLAM techniques, often leading to scale drift and triangulation errors. These limitations collectively impair performance in downstream tasks, including 3D hand and object tracking, human–object interaction recognition, and novel object detection. For instance, benchmarks [14,15] on datasets like Ego4D and HOT3D demonstrate that single-view methods struggle with cluttered backgrounds, self-occlusions, and fast motion, often yielding higher error rates in hand pose estimation and object 6 Degrees of Freedom (DoF) tracking compared to multi-view approaches. In summary, while single-view egocentric systems provide valuable first-person insights, their susceptibility to motion blur, occlusions, and extreme viewpoint variability restricts their reliability in real-world, unconstrained scenarios. These shortcomings have motivated the emergence of egocentric multi-view systems that leverage complementary viewpoints from wearable multi-camera rigs to enhance robustness through geometric consistency and redundancy.

### 1.3. Emergence of Egocentric Multi-View Systems

**Figure 1:**
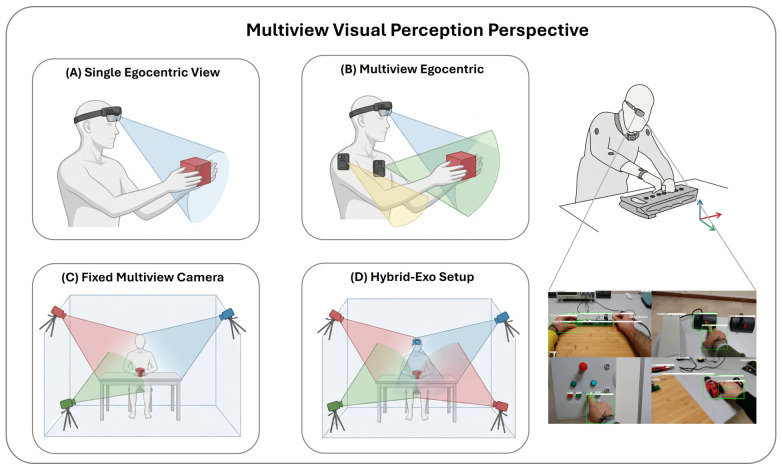
**Comparison of visual perception perspectives.** (**A**) Single egocentric view showing hand–object interaction with typical occlusions and limited field-of-view. (**B**) Multi-view egocentric setup using head-mounted and body-worn cameras for complementary perspectives. (**C**) Fixed multi-camera system in a controlled environment with static geometry. (**D**) Hybrid setup combining first-person and external viewpoints.

**Figure 2:**
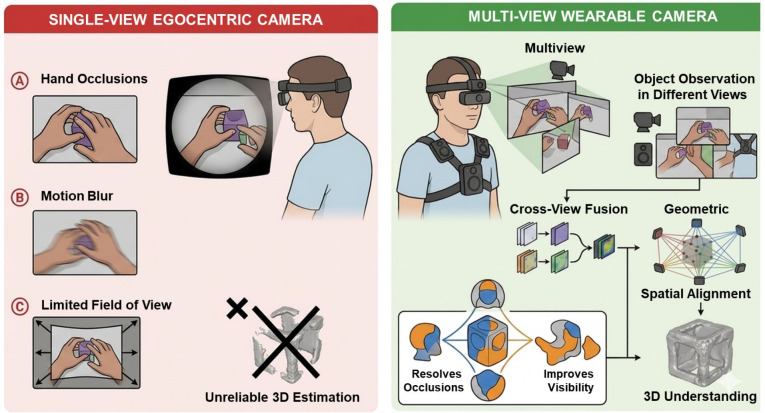
**Multi-view egocentric system advantages.** (**Left panel**): Single-view egocentric camera suffers from hand occlusions, motion blur, and limited field-of-view. (**Right panel**): Multi-view wearable rig (head-mounted plus body-worn cameras) provides complementary observations, enabling robust 3D understanding through cross-view fusion and geometric consistency. Color-coded regions highlight how multiple viewpoints resolve occlusions and improve visibility.

To overcome the inherent limitations of single-view egocentric approaches—such as severe motion blur, frequent self-occlusions, and extreme viewpoint variability—researchers have increasingly turned to egocentric multi-view systems that integrate multiple synchronized cameras into wearable form factors [16,17]. Figure 1 illustrates the evolution from single-view egocentric setups to multi-view configurations, including head-mounted rigs with multiple-angle cameras and body-worn camera arrays. These systems capture complementary viewpoints from the wearer’s perspective, providing redundancy and geometric constraints that enhance robustness in dynamic, open-world scenarios. The emergence of such systems has been driven by advances in hardware miniaturization and the availability of research-oriented wearable platforms. A common example is Meta’s Project Aria glasses–a lightweight AR/AI research prototype equipped with multiple RGB and monochrome cameras, IMUs, gaze tracking, and scene point clouds. When combined with the consumer-grade Meta Quest 3 VR headset, which features dual front-facing cameras, these devices enable synchronized multi-view egocentric capture suitable for real-world deployment. The HOT3D dataset, recorded using both Aria and Quest 3, exemplifies this trend by providing over 833 min (more than 3.7 million images) of synchronized multi-view streams focused on 3D hand and object tracking, demonstrating clear performance gains of multi-view methods over single-view baselines. Beyond head-mounted configurations, custom wearable multi-lens arrays and body-worn rigs have expanded the possibilities. Notable recent developments include the Ego-1K dataset [18], which utilizes a custom head-mounted rig integrating a 4-camera Quest 3 VR headset surrounded by 12 additional synchronized RGB fisheye cameras, yielding nearly 1000 short videos, which convert to approximately 491K frames, tailored for neural 3D video synthesis and dynamic scene understanding. Body-worn multi-camera setups, such as those in the MultiEgoView [16] and MultiEgo [19] datasets, further extend coverage by placing synchronized cameras at multiple body locations (e.g., head, pelvis, wrists, and knees). These rigs leverage GoPro cameras or similar sensors alongside motion-capture suits for ground-truth annotation, enabling improved full-body pose estimation and 4D social scene reconstruction while mitigating occlusions of lower-body parts and distant interactions. The key advantage of these egocentric multi-view systems lies in complementary viewpoints and is illustrated in Figure 2. While a single head-mounted camera often suffers from hand-induced occlusions or limited field-of-view during close-range manipulations, additional cameras positioned around the head or body provide alternative angles that restore visibility [20], improve parallax for depth estimation [21], and enforce geometric consistency across views [22]. This redundancy not only suppresses noise and motion artifacts but also supports advanced techniques such as cross-view feature fusion [23], multi-view stereo [24], and neural rendering for novel-view synthesis in highly dynamic environments [25]. In essence, the shift toward wearable multi-view fisheye lenses and body-worn rigs marks a pivotal evolution in egocentric vision. By leveraging multiple synchronized perspectives, these systems directly address the shortcomings of single-view approaches and lay the foundation for more reliable 3D understanding, human–object interaction modeling, and embodied AI applications in unconstrained real-world settings.

### 1.4. Relation to Broader Multi-View Image Analysis Techniques

Egocentric multi-view image analysis builds upon and extends classical multi-view techniques in computer vision, while introducing unique adaptations necessitated by the dynamic, wearable, and first-person nature of the data. Traditional multi-view image analysis, including multi-view stereo (MVS) [26,27], light-field imaging [28,29], and cross-view fusion [30,31], has primarily focused on static or rigidly calibrated camera setups in controlled environments, such as studio captures or outdoor scenes with fixed baselines. Multi-view stereo aims to reconstruct dense 3D geometry by establishing correspondences across multiple viewpoints and enforcing photometric consistency and epipolar geometry. Classical MVS pipelines [32,33] and learning-based variants [34,35,36] have achieved remarkable success on benchmarks like DTU [37], Tanks and Temples [38], and Middlebury [39]. In egocentric multi-view systems, similar principles are applied but must accommodate continuous camera motion, non-rigid deformations due to head/body movement, and frequent occlusions from hands or close-range objects. Datasets such as HOT3D [6] demonstrate that multi-view egocentric inputs improve 3D hand tracking and 6DoF object pose estimation over single-view baselines by leveraging additional parallax and redundancy, effectively adapting stereo constraints to highly dynamic wearable rigs. Meanwhile, light-field imaging captures the full 4D plenoptic function, such as spatial and angular light distribution, to enable high-fidelity novel-view synthesis and refocusing. While traditional light-field setups [40,41] use dense camera arrays with small baselines, egocentric multi-view systems approximate light-field properties through sparse, wide-baseline wearable cameras (e.g., the 12-camera rig in Ego-1K). This adaptation supports neural rendering techniques such as 3D Gaussian Splatting [42] or NeRF variants [43] for dynamic 4D scene reconstruction, as explored in the MultiEgo dataset for social interactions. However, the large disparities and rapid egomotion in wearable setups introduce challenges unseen in static light-field captures, requiring robust cross-view alignment and motion compensation. Cross-view feature fusion techniques, originally developed for multi-camera surveillance [44,45] or autonomous driving [46,47], align and integrate information across viewpoints to achieve viewpoint-invariant representations. In the egocentric domain, these methods evolve into deformable attention mechanisms and transformer-based architectures that jointly model temporal and cross-view context. For instance, approaches like deformable stereo attention in pose estimation [48] or attention-based blending in single-to-dual-view adaptation [49] exploit epipolar geometry and cross-view consensus to mitigate occlusions and depth ambiguity. The primary distinctions lie in the operational constraints: classical multi-view techniques often assume known, fixed camera calibration and static or slowly moving scenes, whereas egocentric multi-view systems must handle real-time inter-view calibration under continuous motion, severe appearance changes due to egomotion, and open-world variability. This has led to hybrid paradigms that combine classical geometric cues (e.g., epipolar consistency, triangulation) with modern learning-based components (e.g., vision transformers, neural rendering) [50,51] to achieve robust performance in wearable, real-world deployments. In summary, egocentric multi-view image analysis represents a natural evolution of broader multi-view techniques, inheriting core principles of stereo matching, light-field rendering, and cross-view fusion while innovating to address the challenges of mobility, dynamism, and first-person perspective. This synergy directly supports the Special Issue’s focus on techniques in multi-view image analysis in emerging wearable contexts.

### 1.5. Scope and Contributions of This Review

This in-depth review focuses on recent advances in egocentric multi-view image analysis, with emphasis on techniques designed for open-world settings, real-time wearable deployment, and hybrid multimodal understanding. Unlike earlier surveys that treat multi-view aspects only peripherally within broader egocentric vision, this work provides a dedicated and systematic examination of methods that leverage synchronized wearable multi-camera systems to address the unique challenges of first-person, dynamic environments. The scope concentrates on literature published primarily between 2024 and 2026, a period marked by the release of influential multi-view egocentric datasets and associated methodologies. Key resources include HOT3D (2025) with over 833 min of synchronized multi-view streams for 3D hand and object tracking in everyday interaction scenarios [6]; MultiEgo (2025), the first multi-egocentric collection tailored for 4D dynamic scene reconstruction in social interactions [19]; and Ego-1K (2026), featuring nearly 1000 short videos from a custom 12-camera rig for neural 3D video synthesis and dynamic understanding [18]. This temporal boundary is a deliberate scoping choice rather than an oversight of earlier work: the accuracy and stability of stereo-based depth evaluation was already extensively characterized in the classical multi-view stereo literature of the 2000s and 2010s [26,27,52,53,54], discussed further in Section 1.4. Rather than revisiting that early-stage depth-accuracy analysis, this review concentrates on how egocentric wearable constraints—continuous egomotion, non-rigid inter-view baselines, and severe hand occlusion—reshape depth estimation and occlusion handling relative to that classical foundation, which is also why occlusion, rather than raw stereo depth accuracy in isolation, is treated as the central technical thread of this review. These datasets have enabled significant progress in open-world tasks where novel objects, interactions, and environments are common. We prioritize techniques that operate under real-time wearable constraints, including lightweight cross-view feature fusion, efficient geometric consistency enforcement, and edge-optimized neural rendering suitable for power- and thermally limited AR/VR devices. Special attention is given to hybrid understanding approaches that integrate visual cues from multiple egocentric views with other modalities such as motion sensors, gaze tracking, audio, and vision–language models (VLMs) or large language models (LLMs) for enhanced contextual reasoning. The primary contributions of this review are threefold. First, it offers the first dedicated and systematic survey focused exclusively on egocentric multi-view image analysis for wearable multi-camera systems, emphasizing techniques tailored to open-world settings, real-time constraints on edge devices, and hybrid multimodal understanding. Second, it synthesizes the rapid progress enabled by key 2024–2026 datasets—such as HOT3D, MultiEgo, and Ego-1K—while highlighting consistent performance gains achieved through cross-view fusion, geometric consistency, and neural rendering over single-view baselines. Third, this work fills a critical gap in existing literature. Recent comprehensive surveys on egocentric vision [3,8,55] provide excellent overviews of single-view and multimodal egocentric research but treat multi-view techniques only briefly—often as a minor extension or under cross-view understanding subsections. By concentrating on wearable multi-lens arrays and body-worn rigs, this review addresses the growing need for robust, complementary-viewpoint methods that directly mitigate well-documented limitations of single-view approaches in dynamic, real-world scenarios.

## 2. Background and Technical Challenges

### 2.1. Unique Properties of Egocentric Multi-View Data

Egocentric multi-view data exhibit several distinctive properties that differentiate them from both single-view egocentric recordings and traditional fixed-camera multi-view datasets. These properties stem directly from the wearable nature of the capture systems—typically head-mounted or body-worn multi-camera rigs—and the highly dynamic, first-person interaction scenarios in which they are used. A primary characteristic is dynamic inter-view misalignment. Unlike static multi-camera setups where relative camera poses remain fixed, egocentric multi-view systems experience continuous changes in inter-view geometry due to natural head rotations, body movements, and locomotion [55]. This results in time-varying extrinsic parameters, large and rapidly changing baselines, and non-rigid deformations across views. For instance, during hand–object manipulations, a slight head tilt can cause significant shifts in relative camera positions, breaking the assumption of rigid calibration that underpins classical multi-view stereo pipelines. Datasets such as HOT3D [6] and Ego-1K [18] vividly illustrate this challenge, where synchronized multi-view streams must contend with constant egomotion and varying inter-camera relationships. Despite the apparent difficulty of dynamic misalignment, egocentric multi-view data offer substantial redundancy for robustness. Multiple complementary viewpoints provide alternative observations of the same scene element, mitigating the severe self-occlusions, motion blur, and limited field-of-view that plague single-view egocentric systems [56]. When one camera view is heavily occluded by the wearer’s hand or suffers from blur due to fast head motion, other cameras positioned at different angles often capture clearer or more complete information. This redundancy enables more reliable feature matching, depth estimation, and object tracking through cross-view consensus mechanisms. Other unique characteristics of multi-view image analysis include close-range and cluttered interactions. In first-person activities, hands and manipulated objects dominate the foreground, creating highly cluttered scenes with frequent scale variations and extreme appearance changes [57]. The multi-view setup, however, supplies richer parallax cues and multi-angle observations that improve 3D reasoning compared to monocular inputs. Furthermore, the data are inherently multimodal, often accompanied by synchronized IMU measurements, gaze information, and sometimes audio, opening pathways for hybrid fusion strategies [58]. In summary, while dynamic misalignment introduces significant calibration and alignment difficulties, the inherent redundancy and complementary viewpoints of egocentric multi-view data provide a powerful mechanism for overcoming the core limitations of single-view egocentric vision. These properties form the foundation for advanced techniques in cross-view feature fusion, geometric consistency enforcement, and robust 3D understanding in open-world, wearable settings.

### 2.2. Core Technical Challenges

While egocentric multi-view data offer valuable redundancy and complementary viewpoints, they also introduce several formidable technical challenges that must be addressed to realize their full potential in real-world applications. Inter-view calibration and synchronization under head/body motion represent the most fundamental difficulty. In wearable multi-camera systems, relative camera poses change continuously due to rapid head rotations, body movements, and locomotion [59]. Traditional offline calibration techniques, assuming rigid geometry, become unreliable, necessitating online or adaptive calibration methods that can handle non-rigid deformations and time-varying extrinsics. Synchronization across views is equally critical; even minor temporal offsets can break geometric consistency, especially at high frame rates or during fast motion. Datasets such as HOT3D [6] and Ego-1K [18] highlight how imperfect synchronization and motion-induced misalignment complicate stereo matching and 3D reconstruction pipelines. Amplified noise, hand occlusions, and cluttered backgrounds further exacerbate the problem. Close-range first-person interactions cause hands and manipulated objects to dominate the foreground, leading to frequent and severe self-occlusions that are often worse than in single-view setups because multiple cameras may simultaneously capture partial or heavily occluded views of the same interaction. Motion blur from egomotion is amplified across views, while cluttered indoor and outdoor backgrounds introduce significant visual noise and distractors [59]. These factors degrade feature quality and increase the difficulty of establishing reliable cross-view correspondences. The scarcity of large-scale synchronized multi-view egocentric benchmarks has historically slowed the advancement of techniques. Until recently, most egocentric datasets focused on single-view recordings (e.g., Ego4D [4]), with limited multi-view coverage. Although new datasets such as HOT3D [6] (833 min), MultiEgo [19], and Ego-1K [18] have begun to fill this gap, the overall volume and diversity remain insufficient compared to single-view or fixed multi-camera benchmarks. This scarcity limits the training of large-scale models and makes it difficult to evaluate generalization across different wearable configurations and environments. On the other hand, real-time constraints for edge/wearable hardware impose strict limitations on computational complexity, as AR/VR devices and smart glasses operate under tight power, thermal, and memory budgets. Sophisticated multi-view fusion networks, neural rendering, or transformer-based cross-view attention mechanisms must be optimized for on-device inference, often requiring model compression, efficient attention designs, or hybrid edge–cloud architectures. Achieving high accuracy while maintaining low latency, such as 30 frames-per-second (FPS) or higher, remains a significant engineering challenge [60]. Finally, open-world requirements for novel objects and interactions demand robust generalization beyond closed-set training categories. Wearable systems must detect, track, and understand previously unseen objects and HOI patterns in unconstrained daily-life scenarios. This necessitates continual or incremental learning strategies, zero-shot capabilities through vision–language models, and strong regularization from multi-view geometric cues to prevent catastrophic forgetting and improve out-of-distribution performance [61,62]. Addressing these interconnected challenges requires innovations that jointly exploit geometric constraints, cross-view redundancy, and multimodal signals while respecting the practical limitations of wearable deployment.

### 2.3. Comparison with Fixed Multi-Camera and Ego–Exo Setups

Egocentric multi-view systems differ substantially from traditional fixed multi-camera setups and hybrid configurations in terms of camera mobility, geometric constraints, environmental diversity, and practical applicability. Fixed multi-camera systems, commonly used in studio environments or controlled laboratory settings, employ rigidly mounted cameras with static and precisely known relative poses. This rigidity enables straightforward application of classical MVS [34,50], light-field rendering [40,41], and photometric consistency techniques [52,53,54], often yielding high-accuracy 3D reconstructions and dense correspondence. However, such setups suffer from limited mobility and environmental diversity; they are typically confined to indoor scenes with constrained viewpoints and cannot capture natural, unscripted human activities in everyday open-world contexts. The lack of egomotion also means they miss the dynamic, wearer-centric cues essential for understanding first-person interactions, intentions, and hand–object manipulations. In contrast, setups combine one or more wearable cameras with multiple exocentric third-person cameras, as exemplified by Ego-Exo4D [5] and various mobile multi-camera rigs [63,64]. These hybrid systems provide complementary perspectives: the egocentric view captures fine-grained hand–object interactions and the wearer’s immediate field of view, while exocentric cameras offer broader scene context, reduced self-occlusions, and more stable full-body visibility. Ego-exo configurations facilitate cross-view translation, skill learning, and improved 3D hand tracking in diverse environments, including outdoor scenarios. Nevertheless, they require complex synchronization between wearable and stationary cameras, often depend on external infrastructure (e.g., tripod-mounted or backpack-supported exo cameras), and compromise full wearability and untethered mobility. Ground-truth acquisition in datasets can be more challenging in unconstrained settings compared to purely controlled fixed-camera labs. Pure egocentric multi-view systems, such as using Project Aria glasses and Quest 3 with two to three synchronized egocentric views in HOT3D [6], or using multi-egocentric for 4D social reconstruction in MultiEgo [19], and deploying a 12-camera head-mounted rig in Ego-1K, prioritize complete wearability and natural first-person capture. All cameras move together with the wearer, introducing dynamic inter-view misalignment and time-varying extrinsics, but also delivering inherent redundancy through complementary wearable viewpoints without relying on external infrastructure [65]. This design supports true untethered deployment in open-world, daily-life activities while maintaining a strong focus on wearer-centric understanding. The trade-off is increased algorithmic complexity for online calibration, motion compensation, and cross-view fusion under continuous egomotion—challenges largely absent in fixed setups and partially mitigated but not eliminated in hybrids [66,67]. Overall, fixed multi-camera systems excel in geometric precision within controlled environments but lack realism and mobility. Ego–exo setups strike a balance by adding contextual richness at the cost of deployment complexity. Pure egocentric multi-view approaches offer the highest potential for scalable, embodied AI in real-world wearable applications, albeit with the greatest demands on robust dynamic alignment and real-time processing. These distinctions underscore why techniques tailored to egocentric multi-view data—such as deformable attention and hybrid geometric-learning methods—are essential, and require an in-depth review of this emerging field.

## 3. Data Acquisition Systems and Hardware

### 3.1. Head-Mounted Multi-View Rigs

Head-mounted multi-view rigs represent the most common and practical approach for capturing egocentric multi-view data, as they maintain a natural first-person perspective while providing multiple synchronized viewpoints without requiring external infrastructure.

**Figure 3:**
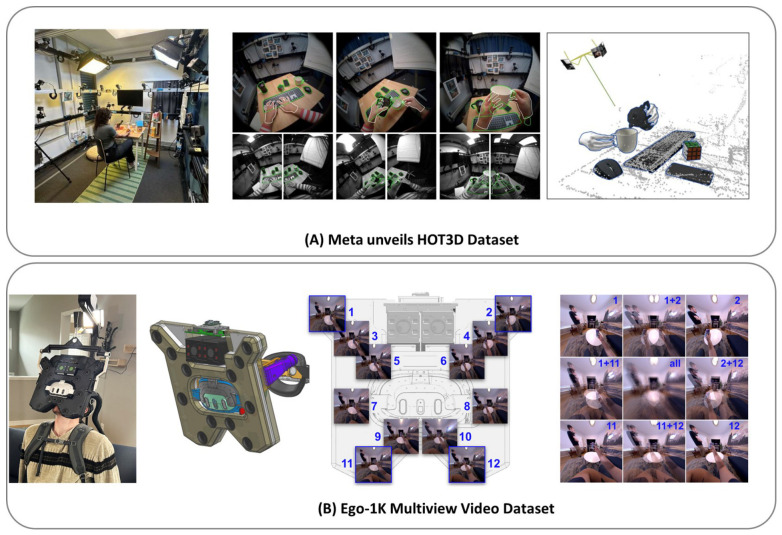
(**A**) Meta Project Aria glasses [7] combined with Meta Quest 3 (HOT3D setup): Aria provides RGB and monochrome cameras with gaze tracking and motion sensors; Quest 3 adds dual monochrome front cameras. (**B**) Ego-1K [18] 12 + 4 camera rig: A 4-camera VR headset core surrounded by 12 synchronized RGB fisheye cameras mounted in a spherical arrangement.

A prominent example is the combination of **Meta’s Project Aria glasses** and **Meta Quest 3 VR headset** used in the HOT3D dataset [6], shown in Figure 3. Project Aria is a lightweight AR/AI research prototype at only 75 g, which is designed for comfortable long-term wear and machine perception. In the HOT3D recording profile, it captures one high-resolution RGB camera (1408×1408 pixels, 110° field-of-view with a fisheye lens) and two global-shutter monochrome cameras (640×480 pixels, 150° field-of-view), along with eye-tracking cameras, dual motion sensors, and scene point clouds from visual odometry. The Quest 3, a widely available consumer VR headset, contributes two monochrome cameras (1280×1024 pixels). All streams are synchronized at 30 FPS, resulting in over 833 min (1.5 million multi-view frames, more than 3.7 million individual images) of hand–object interaction data from 19 subjects interacting with 33 diverse objects.

Another advanced head-mounted configuration appears in the Ego-1K [18] dataset, which employs a custom rig integrating a 4-camera VR headset (based on Quest 3) surrounded by 12 additional synchronized RGB fisheye cameras. This results in a total of 16 cameras, producing 956 short videos (approximately 6.7–9.7 s each, totaling around 491,000 frames) with high-resolution rectified images (1280×1280 pixels, 120° horizontal field-of-view). The dense multi-camera arrangement is specifically designed to support neural 3D video synthesis, dynamic scene understanding, and free-viewpoint rendering from egocentric perspectives. These head-mounted rigs offer several advantages: tight temporal synchronization, co-located sensors for simplified calibration, and natural alignment with the wearer’s gaze and head motion. However, the compact form factor limits camera-to-camera distances, requiring advanced algorithms to handle small parallax in close-range interactions while exploiting redundancy for robustness.

### 3.2. Body-Worn and Multi-User Systems

While head-mounted rigs provide rich first-person views centered on the wearer’s gaze and hand interactions, body-worn multi-camera systems extend coverage to lower-body regions, full-body motion, and multi-user social interactions that are frequently occluded or out-of-view in head-only setups. These configurations typically distribute synchronized cameras across multiple body locations (e.g., head, pelvis, wrists, and knees), offering more complete 360°-like egocentric coverage and enabling advanced applications such as full-body pose estimation, lower-body action recognition, and 4D dynamic scene reconstruction in social contexts. A key example in the multi-user domain is the MultiEgo dataset [19], the first multi-view egocentric dataset specifically designed for 4D dynamic scene reconstruction in real social interaction scenarios. MultiEgo captures synchronized multi-egocentric video streams from multiple participants wearing body-worn or head-mounted cameras during group activities. Recording from several first-person perspectives simultaneously enables reconstruction of dynamic 4D scenes involving multiple interacting humans and objects, supporting tasks such as free-viewpoint video synthesis, holographic documentation of social events, and multi-person human–object interaction understanding. The dataset includes high-fidelity annotations for 3D poses and scene geometry, addressing the limitations of single-egocentric or fixed multi-camera setups in capturing natural, unscripted multi-user dynamics. Complementary to MultiEgo, the MultiEgoView dataset, which is released alongside the EgoSim simulator [16], focuses on body-worn configurations with six synchronized GoPro cameras placed at strategic locations: head, pelvis, left/right wrists, and left/right knees. It comprises 5 h of real-world recordings from 13 participants performing 35 diverse motions (annotated using the BABEL taxonomy [68]), augmented with 119 h of high-fidelity simulated data derived from AMASS motion sequences [69] in virtual environments. Ground-truth full-body 3D poses are provided via an Xsens motion-capture suit, enabling precise evaluation of multi-view pose estimation and motion artifact modeling. This setup is particularly effective for lower-body tracking and activities where head-mounted cameras alone suffer from severe self-occlusion. Body-worn and multi-user systems offer distinct advantages over purely head-mounted rigs: they provide wider spatial coverage, better visibility of the wearer’s own body (especially legs and torso), and richer inter-person geometric constraints for social scene understanding. However, they introduce additional challenges, including more complex inter-camera calibration due to non-rigid body deformation, higher synchronization demands across distributed sensors, and increased data volume. A recurring question for real-world adoption is how far these capture systems can be decoupled from expensive, purpose-built research hardware. MultiEgo already represents a step in this direction: unlike Aria or the Ego-1K rig, it is captured entirely on RayNeo X2 smart glasses, a consumer-grade Android-based AR device with a single 1080p/30 FPS camera and Wi-Fi connectivity, rather than a laboratory prototype. Even lighter-weight and more heterogeneous is EgoKit [70], a 2026 toolkit that extends the same wearable multi-view philosophy to everyday consumer electronics: it exposes a unified egocentric-plus-wrist-view recording workflow across Android phones, iPhones, and consumer XR headsets, pairing any of these host devices with two off-the-shelf USB wrist cameras, a head strap, and a USB-C hub to obtain multi-view coverage without any custom rig or laboratory motion-capture system. This class of low-cost, smartphone-compatible platform is directly relevant to consumer-side deployment, since it trades the sub-millimeter calibration precision of Aria/OptiTrack or dedicated multi-camera rigs for hardware that a wide range of users and research groups already own, making it a useful complement to the higher-precision but higher-cost systems described above when benchmarking real-world, mass-market scenarios.

### 3.3. Sensor Modalities and Synchronization

Egocentric multi-view systems integrate a rich suite of sensor modalities to support robust perception, geometric reasoning, and multimodal fusion. The primary visual modalities include RGB and monochrome cameras. Project Aria glass used for HOT3D [6] consists of one high-resolution rolling-shutter RGB camera (1408×1408 pixels, 110° FOV) that provides color information, while two global-shutter monochrome cameras (640×480 pixels, 150° FOV) deliver high-contrast, low-latency images ideal for tracking and SLAM. The Quest 3 contributes two additional monochrome cameras (1280×1024 pixels). In contrast, MultiEgo [19] employs consumer-grade RGB cameras (1920×1080 at 30 FPS) on RayNeo X2 AR glasses, and Ego-1K [18] uses multiple RGB fisheye cameras in its 12-camera rig. Complementary non-visual modalities enhance temporal and spatial understanding. Inertial Measurement Units (IMUs) provide high-frequency acceleration and angular velocity data (typically dual 1 kHz IMUs in Aria), enabling motion compensation and ego-velocity estimation. Gaze tracking, available in Project Aria via two inward-facing monochrome eye-tracking cameras with IR illumination, delivers per-frame eye-gaze vectors critical for attention modeling, intent prediction, and foveated processing. 3D scene point clouds generated from onboard SLAM further supply dense geometric context for reconstruction and tracking tasks.

Moreover, synchronization and calibration pipelines are critical for maintaining geometric and temporal consistency across heterogeneous sensors. In HOT3D, all image streams, gaze signals, and point clouds are hardware-synchronized at 30 FPS with sub-millisecond accuracy [6]. Concretely, this is achieved via a shared hardware trigger signal. The Aria and Quest 3 sensor pipelines are driven by a common electronic trigger, ensuring that all onboard cameras expose each frame at the exact same physical instant. This approach eliminates the need for post hoc reconciliation of independently free-running clocks. The ground-truth optical marker stream from the external OptiTrack motion-capture system is then aligned to these hardware-triggered image timestamps using a shared SMPTE timecode signal between the recording devices and the mocap system. Intrinsic and extrinsic parameters (including 6DoF transformations between any pair of sensors) are pre-computed during manufacturing and stored in the recording files. For body-worn and multi-user systems such as MultiEgo, a server-client Wi-Fi architecture achieves sub-millisecond temporal alignment using timestamps synchronized to coordinated universal time [19]. This is a fundamentally different, software-based scheme. Instead of sharing a physical trigger line, one smartphone acts as a Wi-Fi hotspot and broadcasts a single start/stop signal to all RayNeo X2 clients over the wireless network. Upon receiving the broadcast, each pair of glasses begins recording independently and timestamps every captured video frame and gyroscope sample using its own local clock, which is disciplined to Coordinated Universal Time (UTC) at 100 ns resolution. Sub-millisecond cross-view alignment is then recovered post hoc by matching these per-device UTC timestamps, rather than being guaranteed at capture time as in the hardware-triggered HOT3D design. Calibration employs structure-from-motion techniques for intrinsic estimation and scene anchoring to fixed objects for consistent coordinate systems. These two schemes represent the two dominant strategies for multi-view egocentric synchronization. Hardware triggering physically enforces simultaneous camera exposure by sharing a common trigger signal. This approach requires devices with exposed trigger lines and is therefore well-suited to single-wearer, laboratory-grade rigs such as an Aria+Quest 3 setup. In contrast, Wi-Fi broadcast combined with precise per-device timestamping relaxes the strict simultaneity guarantee in favor of greater flexibility: it naturally scales to multiple independent, off-the-shelf wearers connected only by a wireless network, at the expense of relying on accurate clock discipline rather than shared hardware.

### 3.4. Ground-Truth Acquisition (Motion-Capture, 3D Scanning)

Accurate ground-truth annotations are essential for training and evaluating egocentric multi-view techniques, particularly for 3D hand and object tracking, pose estimation, and reconstruction tasks. Acquisition pipelines in recent datasets combine professional motion-capture (mocap) systems with high-precision 3D scanning to provide reliable 3D poses, shapes, and meshes while minimizing interference with natural interactions. In the HOT3D dataset, ground-truth acquisition follows a rigorous laboratory protocol [6]. Small optical markers are attached to the participant’s hands and the 33 rigid objects. These markers are tracked using a professional multi-camera mocap system (OptiTrack) installed in a dedicated recording space equipped with infrared cameras and light diffuser panels to handle illumination variations. This setup delivers sub-millimeter accurate 3D poses for hands and objects at high temporal resolution. Hand annotations are provided in two standard formats: UmeTrack and MANO, enabling compatibility with a wide range of hand modeling and tracking methods. For objects, high-fidelity 3D meshes with physically-based rendering (PBR) materials are generated using in-house 3D scanners, capturing detailed geometry and surface properties. The resulting annotations include 3D poses of hands, objects, and cameras, supporting joint hand–object tracking benchmarks. Body-worn and multi-user systems adopt similar strategies with additional considerations for full-body coverage. In MultiEgoView [16], participants wear an Xsens motion-capture suit to obtain precise full-body 3D poses during real-world recordings. Synthetic portions of the dataset leverage AMASS motion-capture trajectories replayed on virtual avatars in Unreal Engine, with simulated sensor noise for realistic evaluation. MultiEgo [19] further extends this to multi-person social scenes, using synchronized mocap data across participants to annotate dynamic 4D interactions and scene geometry. For the Ego-1K [18] dataset, which emphasizes neural 3D video synthesis rather than fine-grained hand–object tracking, the ground-truth focuses on camera calibration and scene consistency. Laboratory calibration with large planar targets provides accurate intrinsics and relative extrinsics, while periodic recalibration during data collection ensures stability over long recording sessions. Scene-level ground truth is often derived from multi-view reconstruction or SLAM outputs rather than marker-based mocap. These acquisition methods ensure high-quality supervision but introduce practical trade-offs. Marker-based mocap requires controlled environments and can slightly alter natural hand movements, while 3D scanning is limited to rigid or semi-rigid objects. Privacy and scalability concerns also arise in multi-user setups [71,72]. Nevertheless, the combination of optical mocap and high-resolution 3D scanning has enabled the creation of benchmarks that reliably demonstrate the superiority of multi-view approaches over single-view baselines in 3D tracking and reconstruction tasks.

## 4. Core Techniques in Egocentric Multi-View Image Analysis

### 4.1. Cross-View Feature Fusion and Geometric Learning

Cross-view feature fusion and geometric learning have been foundational building blocks for effective egocentric multi-view image analysis through different methodologies. A transformer-based architecture that utilizes the fine-grained spatiotemporal correspondence between ego- and exocentric views, together with cross-view attention to align between temporal and spatial features effectively [73]. On the other hand, Liu et al. [74] propose a generative adversarial network (GAN)-based framework to fuse the spatial and temporal attention to transform the appearance between different views. Recently, a robust geometric modeling segmentation framework was proposed [75] with a three-stage architecture to effectively translate high-level feature alignment into a precise segmentation mask. By integrating information from multiple wearable cameras while enforcing geometric consistency, these techniques directly mitigate the dynamic misalignment, occlusions, and noise inherent in first-person data.

#### Multi-View Stereo and Deformable Attention

Multi-view stereo (MVS) and deformable attention mechanisms have emerged as powerful tools for leveraging complementary viewpoints. Traditional MVS pipelines, adapted to wearable rigs, exploit parallax across synchronized views to improve depth estimation and 3D reconstruction, even under continuous egomotion [32,76]. In the HOT3D dataset, multi-view methods outperform single-view counterparts in 3D hand tracking and 6DoF object pose estimation by utilizing additional parallax and redundancy from Project Aria and Quest 3 cameras [6]. Deformable attention further enhances this process by allowing flexible sampling of relevant features across views, dynamically adjusting to varying baselines and motion-induced distortions rather than relying on fixed epipolar geometry [77,78]. To make this geometric constraint modeling explicit, consider two calibrated views. A 3D point X visible in both views must satisfy the epipolar constraint $x_{2}^{⊤}Fx_{1}=0$, where $x_{1}$ and $x_{2}$ are its image projections and F is the fundamental matrix relating the two cameras. Classical wearable multi-view stereo (MVS) pipelines exploit this constraint by sweeping a family of candidate depth planes (or equivalently, by constructing a 3D cost volume indexed by depth hypotheses and per-view matching costs) and then regularizing the volume to recover a dense depth map [32,76]. This formulation assumes that F (i.e., the relative pose between views) is both accurately known and static during matching—an assumption frequently violated by continuous head and body motion. Deformable attention relaxes this rigid requirement by replacing the fixed epipolar search line with a small set of learned sampling offsets around a reference projection. This allows the network to sample slightly off the nominal epipolar line, thereby compensating for pose drift, rolling-shutter distortion, or synchronization jitter [77,78]. Consequently, the hard geometric constraint becomes a soft prior that initializes the attention pattern rather than a strict rule that correspondences must exactly obey. The trade-off is that deformable attention relies on learned weights and provides no closed-form guarantee of geometric consistency. In contrast, plane-sweep or cost-volume MVS strictly respects the epipolar constraint when calibration is accurate, but degrades sharply when it is not. Class-agnostic proposals with epipolar/cross-view consistency address the open-world nature of egocentric interactions. Instead of relying on category-specific detectors, methods generate generic object or hand proposals in one view and propagate them to others via epipolar constraints or learned cross-view consistency losses [79]. This approach improves robustness to novel objects and severe hand-induced occlusions common in close-range manipulations. Multi-view regularization enforces agreement on 3D locations or feature embeddings across cameras, reducing false positives and enhancing proposal quality in cluttered scenes [80]. Transformer architectures for temporal + cross-view context represent the state-of-the-art in unified modeling. These models jointly process sequences across time and views using self-attention or cross-attention layers, capturing both short-term motion dynamics and long-range inter-view dependencies [81]. In dense multi-camera setups such as Ego-1K’s 12 + 4 camera rig, transformer-based fusion enables effective noise suppression, alignment of miscalibrated views, and improved feature representations for downstream tasks like neural 3D video synthesis. Similarly, in MultiEgo’s multi-user social scenes, temporal-cross-view transformers facilitate coherent 4D reconstruction by integrating information from multiple participants’ egocentric streams [82]. Overall, these techniques transform raw multi-view inputs into geometrically consistent, viewpoint-invariant representations. Empirical results on HOT3D, Ego-1K, and MultiEgo consistently demonstrate substantial gains (often 15–30% in key metrics such as MPJPE for hand tracking or PSNR for reconstruction) compared to single-view baselines. Future progress in this area will likely focus on lightweight, edge-optimized variants that maintain high accuracy under strict wearable hardware constraints. Weighing these paradigms against one another, geometric/epipolar-constrained approaches [32,76,79,80] are the most data-efficient and easiest to interpret and debug. Failures can typically be traced to a specific miscalibrated camera pair, but performance degrades sharply when relative-pose estimates drift, a common occurrence under fast egomotion. Deformable cross-view attention [77,78] better tolerates such drift and generalizes more robustly to novel scene content, at the cost of requiring labeled multi-view training data and providing weaker guarantees when the learned offsets are applied far outside the training distribution (e.g., unusually wide or narrow baselines). Generative cross-view translation [74] offers the greatest flexibility for handling large viewpoint changes, including cases with minimal or no visual overlap. However, it is the least geometrically grounded of the three paradigms, and its outputs are not guaranteed to be metrically consistent with the true 3D scene. This limits its applicability to downstream measurement-sensitive tasks such as pose estimation. At the high-compute end of the spectrum, transformer-based temporal-cross-view fusion [73,81,82] achieves the strongest reported results on dense rigs such as Ego-1K and MultiEgo by jointly attending over both time and viewpoints. Its computational cost, however, scales with the number of attended tokens across views and time, making it the least suitable for the real-time, on-device budgets. In practice, the choice among these paradigms is driven less by raw accuracy than by which failure mode (calibration drift, distribution shift, metric inconsistency, or latency) a given deployment can least afford.

### 4.2. Egocentric Multi-View Open-World Object Detection

**Figure 4:**
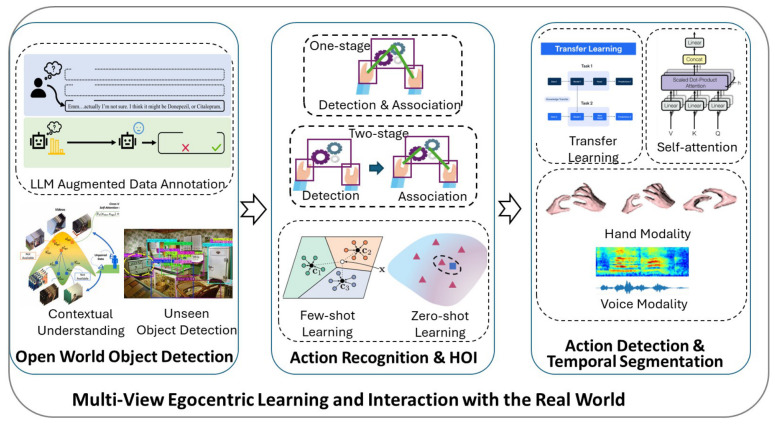
Egocentric multi-view open-world object detection.

Open-world object detection in egocentric multi-view settings requires models to identify, localize, and classify both known and previously unseen objects in highly dynamic, cluttered, and unconstrained environments [42] as shown in Figure 4. Unlike closed-set detection, open-world approaches must handle novel unseen categories, severe occlusions from hands and body parts, and rapid viewpoint changes, making multi-view redundancy and geometric cues particularly valuable [83]. Thus, incremental and continual learning with multi-view regularization has emerged as a key strategy to address catastrophic forgetting and enable lifelong adaptation [62]. In wearable multi-view rigs, these models incrementally update their knowledge as the wearer encounters new objects during daily activities. Multi-view regularization enforces consistency across synchronized camera streams by penalizing discrepancies in predicted 3D locations, feature embeddings, or confidence scores between views. This geometric constraint improves stability during learning and reduces false positives in cluttered scenes. For instance, methods leveraging HOT3D’s synchronized multi-view streams (Project Aria + Quest 3) demonstrate that multi-view regularization boosts robustness in 6DoF object pose estimation for both known and unknown in-hand objects compared to single-view baselines [84,85]. By propagating detections across views via epipolar geometry or learned cross-view attention [86,87], these approaches maintain performance even as the model continually incorporates novel interactions without retraining from scratch.

Large language models (LLMs)-augmented zero-shot annotation and classification further enhance open-world capabilities by leveraging LLMs and vision–language models (VLMs) for semantic understanding without extensive labeled data [88,89]. In egocentric multi-view pipelines, initial class-agnostic proposals generated from one or more views are refined using LLM-guided reasoning. For example, visual features from multiple cameras can be fed into a VLM that describes object appearance, function, or context (e.g., “a cylindrical container likely used for holding liquids”), enabling zero-shot classification of novel items [90]. Multi-view inputs provide richer descriptions by combining complementary angles, reducing ambiguity caused by occlusions or unusual viewpoints. Recent multimodal egocentric models [91,92] (e.g., extensions inspired by MM-Ego and related VLM frameworks) integrate these capabilities, showing improved generalization on datasets like Ego-1K [18] and MultiEgo [19], where social and dynamic scenes introduce diverse novel objects. Together, these techniques enable practical deployment in real-world wearable scenarios, where users continuously encounter new objects and interactions. Multi-view fusion not only supplies additional evidence for detection but also serves as a strong regularizer for continual learning, while LLM augmentation reduces the annotation burden and supports semantic reasoning beyond visual appearance.

These benefits come with limitations that the multi-view setting does not automatically resolve. First, current LVLMs are prone to multi-view hallucination. Park et al. [93] introduced a dedicated benchmark of 4.8k question-answer pairs demonstrating that recent LVLMs such as Qwen2.5-VL and LLaVA-OneVision frequently confuse or mismatch visual evidence across different instances or viewpoints. Simply feeding more camera views into a VLM therefore does not guarantee correct attribution of an observation (e.g., a described handle or label) to the correct object and viewpoint. View ambiguity is thus a measurable and persistent issue rather than an incidental concern. Second, even when a VLM correctly attributes an observation to a specific view, its output is typically a free-text description or class label without an associated 3D pose. Translating a description such as “a cylindrical container likely used for holding liquids” into a metrically accurate and geometrically aligned proposal still requires a separate grounding step, during which any residual cross-view hallucination can compound with grounding errors. Third, VLM/LLM inference is considerably more latency- and memory-intensive than the geometric or attention-based modules described earlier. This creates challenges for sub-30 ms, on-device budgets typical of wearable AR/VR hardware. Most systems cited in this work therefore run the VLM/LLM component off-device or asynchronously, rather than inside the real-time detection loop. Taken together, these hallucination, grounding, and latency limitations indicate that current LLM/VLM augmentation is best viewed as a semantic labeling aid layered on top of a geometry-driven detection pipeline, rather than a drop-in replacement for multi-view geometric verification.

Benchmarks on HOT3D [94,95] highlight clear advantages: multi-view methods achieve superior performance in model-free object detection and 3D lifting of unknown objects, often outperforming single-view approaches by substantial margins in precision and recall under open-world conditions. Future directions include tighter integration of continual learning with real-time edge constraints and more sophisticated LLM-VLM pipelines that actively query the wearer for minimal supervision when encountering highly ambiguous novel objects.

### 4.3. Egocentric Multi-View Human-Object Interaction and Action Recognition

**Figure 5:**
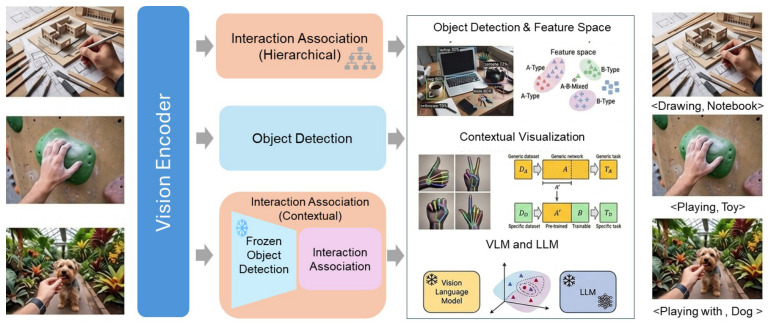
Egocentric multi-view human-object interaction and action recognition.

Video action detection and temporal segmentation in egocentric multi-view settings involve localizing and classifying actions or sub-actions over time while precisely delineating their temporal boundaries in long, untrimmed first-person videos. These tasks are particularly challenging due to rapid viewpoint changes, frequent interruptions, and the fine-grained nature of daily activities. Multi-view systems address these issues by providing complementary temporal observations that enhance both spatial robustness and temporal coherence [55], illustrated in Figure 5. Cross-view attention for noise suppression and alignment has become a central technique for handling the inherent noise and misalignment in wearable multi-view streams. Traditional single-view action detectors suffer from motion blur, hand occlusions, and background clutter that degrade feature quality across frames. In multi-view architectures, cross-view attention mechanisms, which are often implemented via deformable or windowed transformers, selectively aggregate relevant features from different cameras while suppressing unreliable signals [96,97]. For example, when one head-mounted camera experiences severe blur during a quick head turn, attention layers can emphasize clearer views from side or lower-body cameras to maintain consistent action representations. This approach also enforces temporal alignment across views by learning implicit geometric correspondences, reducing the impact of synchronization imperfections. On datasets such as HOT3D [6] and Ego-1K [18], cross-view attention has demonstrated substantial improvements in action detection mAP and boundary precision, particularly for short, transitional actions that are easily fragmented in single-view footage. Multimodal fusion of visual, audio, and hand cues further boosts performance by exploiting complementary information streams available in modern wearable rigs. Visual features from multiple RGB/monochrome cameras capture appearance and motion, while hand skeletons (derived from multi-view 3D tracking) provide precise interaction context. Audio signals, captured via integrated microphones or external wearable recorders, supply semantic cues such as speech, object sounds (e.g., pouring water, typing), and environmental context that are often missing from visual data alone. Effective fusion strategies include late fusion with modality-specific encoders, cross-modal attention, or unified transformer backbones that jointly model visual–temporal, hand-pose, and acoustic features [98,99,100,101]. MultiEgo’s multi-user social scenes [19], multimodal multi-view fusion enables more accurate segmentation of collaborative actions (e.g., passing objects or group conversations) by resolving ambiguities that persist in purely visual pipelines. Recent benchmarks show that combining multi-view visual streams with audio and 3D hand poses yields 10–25% gains in temporal segmentation metrics compared to vision-only single-view methods [101,102]. Overall, the integration of cross-view attention and multimodal fusion transforms egocentric multi-view video into a rich, temporally coherent representation suitable for fine-grained action understanding. These advances are especially valuable for applications requiring precise procedural guidance, such as skill training, assistive technologies, and human–robot collaboration. However, challenges persist in achieving real-time multimodal inference on edge devices and handling noisy or missing modalities in real-world recordings.

The three multimodal fusion strategies surveyed above are not interchangeable. Late fusion with modality-specific encoders [98] is the simplest to implement and the most tolerant of a fully missing modality, as each encoder can be dropped independently. However, because fusion occurs only at the decision or embedding level, it cannot correct errors introduced earlier in a modality’s pipeline (e.g., a mistimed audio event cannot be reinterpreted using visual context). Cross-modal attention [99,100] enables each modality to condition on the others at the feature level. This is particularly effective for resolving ambiguous situations, such as group conversations in MultiEgo, where audio and hand-pose cues disambiguate what vision alone cannot. Its limitation is that it requires reasonably well-synchronized and clean inputs from every modality; otherwise, noise from one modality can propagate into the representations of the others. Unified transformer backbones [101] that jointly model visual–temporal, hand-pose, and acoustic tokens in a single attention stack achieve the strongest reported segmentation gains (10–25% over vision-only baselines). By learning which modality to trust on a per-instant basis, they offer superior performance, but they are the most computationally expensive to train and deploy. Like the transformer-based cross-view fusion, they are the hardest to fit within real-time wearable power budgets. As with the cross-view paradigms above, the practical choice among these fusion strategies is governed less by raw performance than by which failure mode (missing modalities, cross-modal noise propagation, or compute budget) the target deployment can least afford.

### 4.4. 3D Reconstruction, Novel-View Synthesis, and Tracking

3D reconstruction, novel-view synthesis, and tracking represent the highest-level tasks in egocentric multi-view image analysis, transforming synchronized wearable camera streams into spatially and temporally coherent 3D representations of dynamic scenes, hands, and objects. These capabilities are essential for immersive AR/VR experiences, embodied AI, and precise human–robot collaboration. Neural rendering for dynamic 4D scenes has advanced with multi-egocentric datasets such as MultiEgo [19]. By leveraging multiple synchronized first-person views, neural rendering techniques (including variants of Neural Radiance Fields (NeRF) and 3D Gaussian Splatting) could potentially reconstruct temporally consistent 4D scenes involving multiple interacting humans and objects [103,104].

To elaborate on the underlying rendering formalism, NeRF-style methods represent a scene as a continuous function $F_{\Theta }\left(x,d\right)\to \left(c,\sigma \right)$ mapping a 3D position x and viewing direction d to a color c and volume density σ, and render a pixel by integrating this function along its camera ray, $\hat{C}\left(r\right)=\sum_{i}T_{i}\left(1-\exp(-\sigma_{i}\delta_{i})\right)c_{i}$ with accumulated transmittance $T_{i}=\exp\left(-\sum_{j<i}\sigma_{j}\delta_{j}\right)$. 3D Gaussian Splatting instead represents the scene explicitly as a set of anisotropic Gaussians with learned position, covariance, opacity, and color, which are projected and alpha-blended per pixel, trading NeRF’s smooth but slow per-ray sampling for an explicit, sortable primitive that renders at interactive frame rates. Both formulations were originally designed for dense, small-baseline, largely static exocentric captures, so adapting them to egocentric multi-view data requires two further changes: (i) the radiance field or Gaussian set must be extended with a time or deformation variable to model non-rigid scene motion, yielding the dynamic 4D extensions used here, and (ii) because a small number of wide-baseline, rapidly moving wearable cameras provides far sparser angular coverage per timestep than a dense studio rig, photometric and geometric consistency across the available views must be enforced explicitly rather than relying on dense-view redundancy alone [103,104]. This is precisely the role played by MultiEgo’s cross-view consistency terms below, and by egocentric-specific Gaussian Splatting variants such as EgoLifter [42], which are designed to remain stable under the sparse, wide-baseline, egomotion-heavy conditions of wearable rigs rather than the dense, static-camera arrays for which the original formulations were designed.

MultiEgo’s multi-user social interaction recordings enable the modeling of complex dynamics, such as group conversations, object handovers, and collaborative activities, by enforcing cross-view photometric and geometric consistency [19]. These methods support free-viewpoint video (FVV) synthesis from arbitrary virtual cameras, allowing users to replay social events from novel perspectives while preserving realistic motion and appearance. In addition, the complementary nature of egocentric viewpoints helps resolve ambiguities in monocular or single-view neural rendering, particularly for occluded regions and fast-moving elements.

3D hand and object tracking benefits immensely from multi-view redundancy, as demonstrated by the HOT3D benchmark [6]. Using synchronized streams from Project Aria glasses and Meta Quest 3, HOT3D provides large-scale ground-truth annotations for joint hand–object 3D tracking in everyday manipulation tasks. Multi-view approaches outperform single-view baselines by combining parallax cues, cross-view feature fusion, and geometric consistency losses [105]. State-of-the-art methods [106,107] achieve lower mean displacement error for hands, and reduced 6DoF pose estimation errors for objects, even under heavy self-occlusions and rapid motion. Furthermore, the dataset’s 833 min of diverse interactions (kitchen, office, and household activities) with 33 objects highlight the robustness of multi-view tracking in open-world conditions. In-hand object lifting and pressure-aware extensions push the boundaries toward more physically grounded understanding. Once objects are detected and tracked in 3D, lifting techniques reconstruct their full geometry and appearance from partial egocentric observations by integrating multi-view information with shape priors or neural implicit representations. Recent models [108,109] incorporate pressure-aware modeling by fusing visual cues with tactile or force-sensing data (e.g., from instrumented objects or glove-based sensors). These methods estimate contact forces, grasp stability, and pressure distribution during manipulation, enabling finer-grained action understanding and realistic simulation for robotic imitation learning. In multi-view setups, pressure-aware lifting could potentially benefit from more accurate 3D hand poses and object trajectories, reducing ambiguities in contact point estimation. Collectively, these advances demonstrate consistent performance gains of multi-view systems over single-view counterparts across reconstruction fidelity (e.g., higher PSNR and SSIM in novel-view synthesis), tracking accuracy, and physical plausibility. However, challenges remain in achieving real-time 4D rendering on wearable hardware and scaling to longer, more diverse sequences with complex articulations and deformations.

## 5. Datasets and Benchmarks

The rapid progress in multi-view and egocentric visual understanding has been strongly supported by large-scale datasets. These datasets provide geometric supervision, multimodal signals, and cross-view consistency, enabling more robust 3D reasoning and interaction modeling. This section reviews representative benchmarks, summarizes performance trends, and discusses current limitations.

### 5.1. Representative Multi-View and Egocentric Datasets

Table 1 summarizes key datasets, including controlled multi-view capture systems, large-scale egocentric video corpora, and hybrid cross-view benchmarks.

#### 5.1.1. Geometrically Grounded Multi-View Datasets

One of the earliest multi-view datasets, the CMU Panoptic Studio, introduced a dense multi-camera system with more than 480 cameras. It enables accurate 3D human pose reconstruction and social interaction analysis [110], providing high geometric precision; however, its strength is limited due to the indoor laboratory environments. For hand-centric analysis, InterHand2.6M contains 2.6 million frames of interacting hands captured using multi-view cameras with accurate 3D annotations [111], improving the robustness to self-occlusion and inter-hand contact. Besides, Kwon et al. proposed H20, which focuses on two-hand manipulation using multi-view RGB-D data and supports fine-grained hand–object interaction modeling [112]. Most recently, HOT3D extended multi-view supervision to hand–object tracking, providing 833 min of synchronized recordings with precise joint and object pose annotations [6].

#### 5.1.2. Large-Scale Egocentric Datasets

In contrast to controlled multi-view systems, large-scale egocentric datasets emphasize real-world diversity. EPIC-KITCHENS-100 [113], an extension from the popular EPIC-KITCHENS dataset [114], contains over 100 h of first-person kitchen activities and supports action recognition and anticipation, but the 2D-only annotations limit its usefulness. Ego4D [4] represents a major milestone, offering more than 3000 h of egocentric video collected worldwide. It includes multimodal signals such as audio, eye gaze, stereo, together with synchronized videos from multiple egocentric cameras, and IMU data, supporting tasks including action recognition, temporal localization, and anticipation.

Building on this, Ego-Exo4D [5] provides synchronized egocentric and exocentric views, enabling cross-view reasoning and 3D consistency modeling. Finally, MultiEgo [19] further explores 4D human reconstruction by combining first-person and external viewpoints for dynamic 3D modeling.

### 5.2. Performance Trends: Multi-View vs. Single-View

In this section, results from recent literature are presented for both single-view and multi-view approaches on various tasks. Across benchmarks, multi-view supervision consistently improves performance and proves its superiority in terms of geometric accuracy and robustness to occlusion.

#### 5.2.1. 3D Hand Pose Estimation

For the 3D hand pose estimation task, we surveyed methods on InterHand2.6M [111]. From Table 2, it is evident that multi-view approaches consistently outperform their single-view counterparts in terms of geometric accuracy. Specifically, multi-view approaches such as Feng et al. [115] and Han et al. [116] achieve significantly lower Mean Per Joint Position Error (MPJPE) and Mean Per Vertex Position Error (MPVPE) at around 30% compared to single-view methods. This performance gap validates the premise that by utilizing the cross-view constraints, one can significantly reduce depth ambiguity and object occlusion, limiting 3D pose errors. In addition, by leveraging temporal data in [117,118], single-view methods can yield a performance boost over static single-frame approaches, as temporal convolution and SLAM-based infilling can mitigate frame-level occlusions that classic transformers or feature fusion can not handle.

Mechanistically, this performance gap arises because InterHand2.6M’s two-hand interactions constitute the regime in which a single camera is most geometrically ill-posed. When one hand occludes part of the other, or when a hand is viewed nearly edge-on, monocular methods must rely entirely on learned shape priors to infer the missing geometry. In contrast, multi-view methods such as those by Feng et al. [115] and Han et al. [116] can triangulate occluded joints from a second, unoccluded view. Even the best multi-view method still exhibits a residual error of 5.65 mm MPJPE. According to the failure-mode analyses reported in this line of work, this error is concentrated in two challenging cases: (1) configurations in which both hands are heavily occluded from all available viewpoints simultaneously (e.g., tight in-hand grasps), and (2) situations where inter-hand contact makes individual joint correspondences ambiguous regardless of the number of cameras. In short, multi-view redundancy effectively resolves single-view occlusions but cannot overcome occlusions that are shared across all views. This also explains why temporal single-view methods [117,118] partially close the gap without adding cameras: they substitute redundancy across time for redundancy across viewpoints. This approach helps with transient occlusions but remains limited for persistent ones that last for the full duration of an interaction.

#### 5.2.2. Hand–Object Interaction

For the Hand-Object Interaction task, HOT3D shows that multi-view egocentric inference improves 3D hand–object tracking compared with monocular baselines. In Table 3, multi-view hand tracking reduces the Mean Keypoint Position Error (MKPE) by 27.2–29.2% when using the UmeTrack [123] baseline, and multi-view 6DoF object pose estimation improves the Recall at 10 cm (R@10) by 20.0–26.9% with the FoundPose [124] baseline across Quest 3 and Aria devices. For the in-hand object lifting task, the performance gained by applying the multi-view stereo matching method (StereoMatch) is even larger compared to the single-view monocular depth approach (MonoDepth) with R@10 gains of 142.1–185.4% using both ground truth (GT) and predicted masks on the Aria device.

The especially large gain for in-hand lifting (142–185%) relative to hand tracking (27–29%) and object pose (20–27%) is explained by task geometry rather than by the stereo-matching method per se. Hand tracking and object pose only need to localize a small number of joints or a single rigid pose, for which even one noisy view often provides a usable, if biased, estimate, giving MonoDepth and single-view UmeTrack a non-trivial baseline to improve upon; in-hand lifting instead requires recovering dense, per-pixel depth of a mostly hand-occluded object at close range, a regime where a single camera frequently has no unoccluded line of sight to large parts of the object surface at all, so MonoDepth’s baseline recall (23.3–30.2) sits close to a floor that any usable second viewpoint improves upon dramatically. Based on the failure modes reported in this line of work, multi-view stereo matching still under-performs on lifts where the object is almost entirely enclosed by the hand, leaving too little visible surface in any view to anchor a match, and on thin or reflective objects, where even correctly triangulated points yield noisy or specular stereo correspondences.

Table 3 also lets us isolate the effect of view *count* directly, since the same tasks are evaluated on both the 2-view Quest 3 rig and the 3-view Aria rig. For 6DoF object pose, the extra view provides a modest additional benefit (26.9% gain for Aria’s three views versus 20.0% for Quest 3’s two), consistent with diminishing returns once a second view has already resolved most depth ambiguity. For in-hand lifting with ground-truth masks, however, Quest 3’s 2-view setup reaches a higher absolute recall (96.8) than Aria’s 3-view setup (86.2), which is counter-intuitive if one assumes that more views are always better; the likely explanation is that Quest 3’s two cameras are positioned specifically to maintain stereo overlap on the region directly in front of the headset where in-hand manipulation occurs, whereas Aria’s third camera trades some of that targeted overlap for a wider field of view better suited to scene-level tasks. This illustrates a broader trade-off that recurs across the rigs surveyed in Section 3: 2-view stereo pairs (Quest 3; MultiEgoView’s paired limb cameras) are the cheapest to synchronize and calibrate and suffice when the two views are placed to guarantee overlap on the region of interest; 3-view head-mounted rigs (Aria + Quest 3 in HOT3D) add modest accuracy and robustness to the occlusion of any single view at a small increase in calibration and bandwidth cost; and dense 12–16-view rigs (Ego-1K) are necessary for full free-viewpoint neural rendering, but at a computational and storage cost is not currently compatible with real-time on-device inference. Selecting a configuration is therefore a matter of matching camera count and placement to the task’s geometric requirement, rather than simply maximizing the number of views.

#### 5.2.3. Action Recognition and Anticipation

Table 4 summarizes the anticipation results under the standard single-view settings for Ego4D [4]. For long-term action anticipation (LTA), recent VLM/LLM-based methods [125,126] improve over the original TimeSFormer [127] baseline, reducing action edit distance from 0.9253 to 0.8503. For short-term object interaction anticipation (STA), gains are mainly obtained by incorporating stronger temporal and action-context modeling [128], with overall full action mAP improving from 3.61 to 5.18. These results show that Ego4D primarily reflects progress in monocular egocentric representation learning, multimodal reasoning, and language-guided context modeling, rather than explicit multi-view inference due to the dataset characteristics. Therefore, Ego4D serves as a useful single-view reference point, while direct single-view versus multi-view analysis is better supported by Ego–Exo4D [5].

In the next application, Table 5 demonstrates that the benefit of multi-view learning on Ego–Exo4D is task- and method-dependent. For keystep recognition, naive training does not always help: TimeSFormer [127] drops from 35.24 to 29.84 accuracy, suggesting that simply adding exocentric supervision can introduce viewpoint mismatch. In contrast, EgoVLPv2 [129] and GLEVR [130] obtain modest gains, indicating that cross-view information is more useful when handled through view-aware pretraining, and graph-based alignment improves the accuracy by a significant margin (38.69% vs. 53.08%).

**Table 4:** Single-view anticipation results on Ego4D [4].

Model	Input	Metric	Result
*Long-term Action Anticipation (LTA)*			
Bertasius et al. [127]	Ego RGB	${\mathrm{ED}}_{V/N/A}\;↓$	0.7169/0.7359/0.9253
Mittal et al. [125]	Ego + VLM	${\mathrm{ED}}_{V/N/A}\;↓$	0.679/0.681/—
Kim et al. [126]	Ego + VLM/LLM	${\mathrm{ED}}_{V/N/A}\;↓$	**0.6471**/**0.6117**/**0.8503**
*Short-term Object Interaction Anticipation (STA)*			
Bertasius et al. [127]	Ego RGB	${\mathrm{mAP}}_{N/NV/NT/A}\;↑$	26.15/9.45/8.69/3.61
Ragusa et al. [131]	Ego RGB	${\mathrm{mAP}}_{N/NV/NT/A}\;↑$	25.06/13.29/9.14/5.12
Pasca et al. [128]	Ego + Action Context	${\mathrm{mAP}}_{N/NV/NT/A}\;↑$	**30.43**/**13.45**/**10.38**/**5.18**

*Note:* Ego4D results are primarily single egocentric-view settings. **Bold** indicates the best results. —denotes no available results. ED denotes the normalized edit distance for verb (V), noun (N), and action (A). STA mAP is reported for noun (N), noun-verb (NV), noun-TTC (NT), and full action (A).

**Table 5:** Single-view and multi-view analysis on Ego-Exo4D [5].

Model	Setting	Metric	Single	Multi	Gain
*Keystep Recognition*					
Bertasius et al. [127]	Ego vs. Ego + Exo train	Acc. ↑	35.24	29.84	−5.40%
Pramanick et al. [129]	Ego vs. Ego + Exo	Acc. ↑	37.85	38.69	2.2%
Romero et al. [130]	Ego graph vs. Ego + Exo graph	Acc. ↑	**52.36**	**53.08**	+0.72%
*Proficiency Estimation*					
Bertasius et al. [127]	Ego vs. Ego + Exos	Acc. ↑	42.3	40.8	−1.5%
Bianchi et al. [132]	Ego vs. Ego + Exos	Acc. ↑	45.9	47.5	+1.6%
Bianchi et al. [133]	Ego vs. Ego + Exos	Acc. ↑	**47.3**	48.0	+0.7%
Bianchi et al. [134]	Ego vs. Ego + Exos	Acc. ↑	44.2	**48.2**	+4.0%
Tanoue et al. [135]	Ego vs. Ego + Exos	Acc. ↑	44.3	47.8	+3.5%
Braun et al. [136]	Ego vs. Ego + Exo + HR	Acc. ↑	39.69	43.94	+4.25%

*Note:* “Single” denotes ego-only input unless otherwise stated. “Multi” denotes Ego+Exo, except Braun et al. [136], which additionally uses estimated heart-rate features. **Bold** indicates the best results.

For proficiency estimation, multi-view fusion is more consistently beneficial. Recent methods improve over ego-only baselines by roughly 0.7–4.25 percentage points, with ProfVLM [134] achieving the best multi-view accuracy of 48.2%. These results suggest that exocentric views provide complementary cues for assessing body motion, execution quality, and scene context, while egocentric views remain crucial for close-range hand–object details. Overall, Ego-Exo4D supports that multi-view information can improve fine-grained activity understanding, but the gain depends strongly on the task formulation.

The negative result for naive training on keystep recognition is worth explaining mechanistically rather than only reporting it as an exception. TimeSFormer processes ego and exo frames with the same spatiotemporal patch-attention weights, so when exocentric frames are simply concatenated into the input without any mechanism to indicate which viewpoint a patch came from, the model must generalize its ego-tuned attention patterns to a visually very different, third-person distribution; this acts as a source of distribution shift rather than a source of useful redundancy, making it a genuine failure case of multi-view fusion rather than merely a smaller gain. EgoVLPv2 and GLEVR avoid this failure by giving the model an explicit signal about view identity, either through a view-aware pretraining objective or through a graph structure that keeps ego and exo features as distinguishable nodes rather than merging them into one stream, which is why they see gains instead of degradation, and why GLEVR’s graph-based alignment reaches the highest multi-view accuracy of the three methods (53.08% versus EgoVLPv2’s 38.69%). Proficiency estimation, in contrast, is helped consistently because the task itself, assessing execution quality and body-motion correctness, depends on information such as full-body posture and distance to the workspace that is often outside an egocentric camera’s field of view by construction, so exocentric input supplies genuinely complementary evidence rather than a redundant or conflicting view of the same content; this is also why the largest proficiency gain in Table 5 comes from adding a physiological exocentric signal (heart rate) rather than another visual viewpoint [136]. Read together, the keystep and proficiency results indicate that multi-view fusion helps reliably when the additional view supplies information the primary view structurally cannot capture, and helps unreliably, or actively hurts, when it merely duplicates the primary view’s content without an explicit mechanism to reconcile the two.

#### 5.2.4. Consolidated Meta-Analysis of Multi-View Performance Gains

The individual task analyses above each cite a gain figure of their own, and the abstract summarizes these collectively as a 15–30% range; Table 6 consolidates every quantitative multi-view-versus-single-view comparison reported in this review into one place so that the true spread behind that summary figure, rather than only its central tendency, is visible.

Three observations follow from this consolidated view. First, the 15–30% headline figure is a reasonable summary of the geometry-dominated tasks (hand pose, hand tracking, object pose), where triangulation from a second view directly resolves the depth or occlusion ambiguity that limits single-view performance. Second, tasks that are closer to semantic recognition than to geometric estimation show a much wider and occasionally negative range: naive fusion for keystep recognition can lose 5.4 accuracy points relative to an ego-only baseline, and even the best view-aware method on this task gains less than one point, which is an order of magnitude smaller than the geometric-task gains. Third, in-hand object lifting is a clear outlier on the high end (142–185%) precisely because, as discussed above, its single-view baseline is closest to a geometric floor rather than a competitive baseline. We therefore report this meta-analysis, rather than a single aggregate percentage, as the more accurate summary of what multi-view supervision buys across tasks: substantial and reliable for tasks bottlenecked by geometric ambiguity, and modest, method-dependent, or occasionally negative for tasks bottlenecked by semantic recognition or by naive (view-unaware) fusion design.

### 5.3. Limitations and Research Gaps

Despite substantial progress, several limitations remain. **Indoor bias:** High-precision multi-view datasets such as Panoptic Studio [110], HOT3D [6], and InterHand2.6M [111] are mainly collected in controlled indoor environments. In-the-wild and socially complex scenes are underrepresented and mainly addressed by single-view egocentric datasets such as Ego4D [4] and EgoCampus [137]. This limits the generalization of multi-view methods to real-world scenarios with diverse lighting, backgrounds, and social interactions.

**Scale versus precision trade-off:** Large-scale datasets such as Ego4D [4] offer environmental diversity but limited 3D ground truth. In contrast, geometry-focused datasets [111,112] provide accurate annotations but limited real-world variation.

**Limited multi-person manipulation:** Most hand–object benchmarks focus on single-user interactions, such as EPIC-KITCHENS-100 [113], or Ego4D [4]. Collaborative or competitive multi-person manipulation scenarios remain insufficiently studied. On the other hand, datasets like InterHand2.6M [111] focus on two-hand interactions but do not capture multi-person dynamics, thus limiting the development of frameworks that can handle complex social interactions involving multi-person interactions.

**Sensor generalization:** Many datasets rely on calibrated, high-end capture systems, reducing transferability to consumer devices such as smartphones or AR headsets. Panoptic Studio [110] and InterHand2.6M [111] rely on multiple synchronized cameras in controlled settings, while HOT3D [6] uses either Quest 3 or Aria glasses, which may not be available to the broader research community. In addition, these datasets rarely introduce severe motion blur, auto-exposure lag, or shutter effects compared to the common consumer devices, which may lead to a performance drop when applying the trained models to real-world applications.

**Standardized cross-modal evaluation:** Although multimodal streams (audio, IMU, gaze) are available, unified benchmarks for evaluating cross-modal fusion methods remain limited. Ego4D [4] and Ego–Exo4D [5] focus on action anticipation, keystep recognition using metrics like mAP or F1-scores, while other tasks, such as hand pose estimation and hand–object tracking, rely on geometric metrics (MPJPE, R@10). Thus, by standardizing the evaluation metrics and modalities across modalities and tasks, a more comprehensive and objective benchmark can be established.

## 6. Applications and Broader Impact

**Figure 6:**
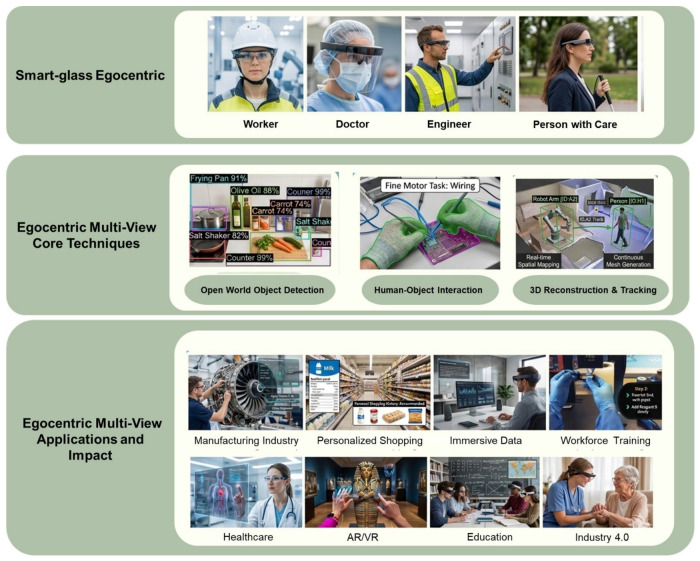
Applications of multi-view image analysis, egocentric perception, and cross-view learning in AR/VR, healthcare, and Industry 4.0 transformation.

Advances in multi-view image analysis, egocentric perception, and cross-view learning have enabled important applications across many domains. By combining geometric consistency, multimodal fusion, and spatial–temporal reasoning, these systems move beyond laboratory research into real-world social and economic settings, especially in AR/VR, robotics, healthcare, manufacturing, and social applications as illustrated in Figure 6.

Accurate 3D hand tracking and scene reconstruction allow natural interaction in head-mounted displays and spatial computing platforms. Multi-camera systems improve robustness under occlusion, which is essential for gesture control and object manipulation in immersive environments [123]. In robotics, multi-view analysis [6,138] supports 3D pose estimation, grasp detection, contact modeling, and dynamic scene understanding. By reducing depth uncertainty—especially in cluttered scenes—multi-view geometry improves reliability in robotic manipulation. Furthermore, in human–robot collaboration (HRC), cross-view reasoning helps robots interpret human intent through body pose, gaze direction, and hand movement [139], which shared understanding improves coordination and safety in collaborative workspaces. Recent research [5] also combines egocentric and exocentric views, allowing robots to align their internal scene representation with a human partner’s perspective, improving task efficiency and shared autonomy. Because AR/VR headsets and mobile robot platforms operate on tight power and thermal budgets, edge deployment here is a first-order design constraint rather than an afterthought: the cross-view fusion and neural-rendering components described in Section 4.4 must run within the low-latency budgets discussed in Section 7, which is why several of the pipelines cited above favor lightweight attention or on-device SLAM over the larger transformer backbones used for offline benchmarking.

A related but structurally distinct application domain is Advanced Driver-Assistance Systems (ADAS). In ADAS, multiple fisheye or wide-angle cameras are mounted around a vehicle to construct a surround, bird’s-eye-view representation for parking assistance, blind-spot monitoring, and open-world obstacle detection [140]. This domain shares much of the core machinery surveyed in this review, including multi-camera cross-view fusion, fisheye geometric calibration, and open-world detection in highly dynamic environments. We nonetheless treat ADAS as adjacent to, rather than within, the scope of this review. The cameras are rigidly mounted to the vehicle chassis, so their relative geometry is fixed and classical multi-camera calibration applies directly. Moreover, the system does not experience the continuous, non-rigid inter-view motion (Section 1.3) that characterizes the egocentric setting, even though the vehicle itself moves through an open, uncontrolled world. In this sense, the architectures illustrated in Figure 1C,D are closer in spirit to this vehicle-mounted, fixed-baseline paradigm than to the wearable systems that are the primary focus of this review. We highlight the connection here because the cross-view fusion and open-world detection techniques discussed in Section 4.1 and Section 4.2 transfer substantially to this adjacent, highly active application area.

In healthcare and assisted living, multi-view and egocentric systems support rehabilitation, monitoring, and assistive technologies. Accurate 3D pose tracking enables objective assessment of motor recovery after stroke or injury, reducing measurement errors compared to single-camera systems [141,142]. Besides, in elderly care, multi-camera smart-home setups [143] improve fall detection by overcoming occlusion and limited viewpoints, and temporal reasoning across views also supports early detection of abnormal gait patterns. Moreover, egocentric wearable systems assist visually impaired users through scene understanding, object localization, and activity summarization, especially when vision is combined with audio signals [144,145,146]. For the telemedicine task, multi-view and multimodal capture provide clinicians with more reliable motion feedback than standard video calls [147]. Nevertheless, healthcare deployment requires strong privacy protection, secure data management, and compliance with regulatory standards, particularly for continuous monitoring systems. In practice, this favors on-device pose estimation and on-device de-identification (e.g., face blurring performed before any frame leaves the device) over cloud offloading. Continuous multi-camera monitoring of patients or elderly residents is precisely the scenario in which transmitting raw video creates the greatest privacy risk. In addition, the extra cameras and compute required for multi-view coverage increase the per-bed or per-room deployment cost compared to a single fixed camera. This represents a practical adoption barrier in resource-constrained clinical and home-care settings, even where the accuracy benefits of multi-view are most compelling.

In manufacturing and industrial settings, multi-view image analysis further enhances automation, safety, and workforce training. Multi-camera systems improve precision in assembly lines by enabling accurate part alignment, micro-defect detection, and real-time verification of production stages [148,149]. Moreover, cross-view geometric consistency reduces false detections caused by reflections or occlusions [150]. Besides, egocentric recordings of skilled workers support learning-from-demonstration frameworks, enabling knowledge transfer to robots or novice trainees [151,152]. By utilizing the multi-view replay, detailed observation of hand movements from different angles can be recorded and reviewed, accelerating skill acquisition. In addition, multi-view tracking provides objective productivity and ergonomic measurements, potentially reducing repetitive strain injuries [153]. Nevertheless, workplace deployment must ensure transparency, fairness, and informed consent when monitoring technologies are used. From a cost-control perspective, the additional cameras, synchronization hardware, and compute required for multi-view coverage must be weighed against the defect-detection or ergonomic gains they provide at each station. Industrial adopters therefore typically instrument only the highest-value or highest-risk stations first, rather than an entire production line. Running the resulting cross-view fusion models at the edge, directly on line-side hardware, is also generally preferred over cloud processing. The latency and connectivity dependence of cloud offloading are difficult to reconcile with the real-time verification and safety requirements of a factory floor.

Multi-view systems also contribute to education, entertainment, and cultural preservation. In education, 3D reconstruction enables virtual laboratories where students interact with objects using realistic spatial feedback [154]. Egocentric analytics can help instructors better understand student attention and engagement in both online and offline learning environments [82,155]. In entertainment and gaming, multi-view motion capture improves realism in film production and interactive media, and real-time volumetric capture allows users to appear inside virtual environments with full-body and hand tracking [156]. For cultural heritage preservation, multi-view 3D reconstruction supports the digital archiving of artifacts, performances, and traditional craftsmanship, and egocentric recordings help preserve practical knowledge that might otherwise disappear [91,157]. These lower-stakes settings still impose meaningful practical constraints. Classroom and venue deployments are typically far more cost-sensitive than clinical or industrial ones, which favors the lightweight, consumer-grade capture platforms discussed in Section 3.2 over research-grade rigs. In addition, recording students, performers, or visitors again raises the same on-device privacy processing considerations highlighted in the healthcare context above.

To summarize, the broader societal and economic impact of multi-view image analysis is significant and will continue to grow further. Applications in robotics, AR/VR, and smart manufacturing support Industry 4.0 transformation, improving productivity and reducing operational costs. These systems increasingly act as augmentation tools rather than replacements for workers, enhancing safety, reducing physical strain, and improving training efficiency. Assistive technologies based on egocentric perception promote accessibility and independence for individuals with disabilities in the health sector. Multi-view systems can also enhance public safety through improved crowd monitoring, emergency response coordination, and infrastructure inspection. At the same time, widespread deployment raises ethical and privacy concerns, including risks of surveillance, data misuse, bias, and unclear data ownership. Nevertheless, responsible AI frameworks, privacy-preserving methods such as on-device processing and federated learning, and transparent governance mechanisms are essential to ensure sustainable and socially beneficial adoption.

## 7. Open Challenges and Future Directions

**Figure 7:**
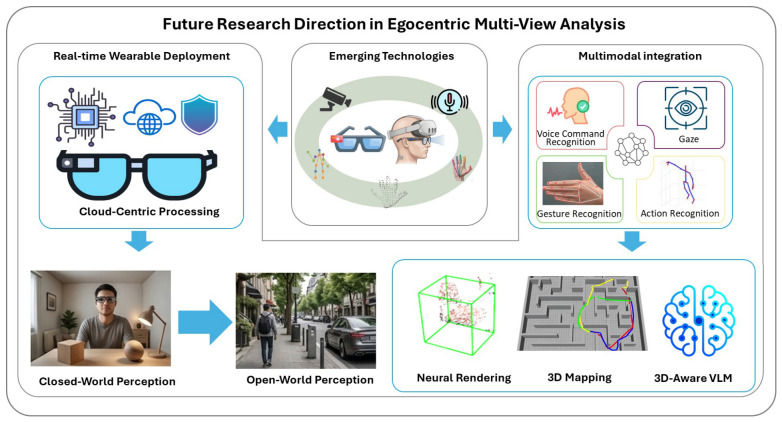
Future research directions in egocentric multi-view analysis.

Despite rapid progress in multi-view perception, egocentric vision, and 3D-aware learning, several important challenges remain and are visualized in Figure 7. These challenges involve computational efficiency, dataset scalability, multimodal integration, evaluation standardization, and generalization to open-world environments. A major limitation for real-world deployment is the high computational cost of multi-view fusion. Many existing approaches rely on dense feature matching [119], volumetric reconstruction [121], or transformer-based cross-view attention [120,127], which require significant memory and processing power. However, wearable platforms such as AR glasses and mobile robots demand low-latency inference (e.g., below 30 ms) while maintaining spatial consistency across views. Achieving real-time multi-view fusion under dynamic camera motion and partial visibility remains an open challenge. Promising directions include lightweight backbone networks, sparse or window-based attention mechanisms, model compression, and efficient neural rendering techniques. Another key issue is the need for large-scale, diverse, and privacy-preserving benchmarks. Although recent datasets [4,5] have expanded the scale of egocentric video collection, limitations remain in outdoor coverage, multicultural contexts, and long-tail activities. Continuous first-person recording [6] also raises serious privacy concerns. Thus, future benchmarks should incorporate on-device anonymization, face and object blurring protocols, federated data collection strategies, and consent-aware data pipelines. Synthetic data generation and simulation platforms can help increase diversity while reducing privacy risks, but ensuring realistic transfer to real-world environments remains challenging. Full multimodal integration is another open problem. Modern datasets [5,6] often include vision, audio, IMU, and gaze signals, yet many models still process these modalities independently or combine them using simple late-fusion strategies. Next-generation systems should support precise temporal alignment across sensors, deep cross-modal reasoning, and robustness to missing inputs. Besides, recent progress in multimodal large language models and vision–language models [125,126] suggests a pathway toward language-guided embodied perception, where language serves as a reasoning interface over multimodal streams. However, grounding language in accurate 3D geometry and physical interaction is still unresolved. Most current systems are trained in closed-world settings with fixed categories and predefined tasks [18,113]. In contrast, real-world agents must operate in dynamic and continuously evolving environments. Key research challenges include continual learning without catastrophic forgetting, open-vocabulary object and action recognition, and adaptation to unseen environments.

Future research topics might also include embodied intelligence, which aims to integrate perception, planning, and control into a unified framework for multi-view systems. Combining reinforcement learning in simulation with real-world multi-view data is promising, yet sim-to-real transfer remains difficult due to appearance, dynamics, and sensor-domain gaps [16,158]. In addition, hybrid egocentric–exocentric learning is also emerging as a powerful direction, with Ego–Exo4D [5] showing the value of synchronized first-person and third-person observations for skilled activity understanding. Recent cross-view translation methods further explore ego–exo alignment and view synthesis, including exocentric-to-egocentric video generation [74,159], offer promising tools for consistent multi-view reconstruction and novel-view synthesis. Finally, the lack of standardized evaluation protocols is another challenge that needs to be addressed. Benchmarks such as HOT3D [6], Ego4D [4], and Ego–Exo4D [5] demonstrate the importance of public evaluation tasks and open challenge servers, but metrics remain fragmented across 3D pose, hand–object interaction, reconstruction, and action understanding. Future research should define more unified multi-view benchmark suites, standardized multimodal evaluation metrics, cross-dataset generalization protocols, and open evaluation servers to improve reproducibility and transparency.

## 8. Conclusions

Egocentric multi-view image analysis has emerged as a pivotal paradigm for robust perception in open-world, mobile environments. By leveraging synchronized wearable cameras, these systems overcome fundamental limitations of single-view egocentric vision—such as occlusion, motion blur, and restricted field-of-view—as well as the environmental rigidity of traditional fixed multi-view setups. This review has systematically examined recent advances in cross-view feature fusion, geometric consistency learning, human–object interaction modeling, action understanding, 3D reconstruction, and novel-view synthesis tailored to wearable multi-camera systems. We highlighted a new generation of large-scale datasets released between 2024 and 2026, including HOT3D, MultiEgo, Ego–Exo4D, and Ego-1K, which collectively push the boundaries of multi-view egocentric understanding in terms of scale, modality richness, and spatial-temporal alignment. When combined with advances in vision–language models and large language models, these datasets have enabled consistent performance gains over single-view baselines across key tasks. Beyond methodological progress, we emphasized the strong alignment of this research direction with emerging themes in multi-view stereo, cross-view learning, and neural rendering-based novel-view synthesis. Importantly, egocentric multi-view systems are increasingly integrated into higher-level reasoning frameworks that support embodied intelligence and multimodal decision-making. The societal implications are substantial, with emerging applications in immersive AR/VR, assistive robotics, healthcare monitoring, rehabilitation, and human-computer interaction highlighting the transformative potential of wearable multi-view perception systems. However, several critical challenges remain unresolved, including robust calibration under unconstrained motion, limited benchmark diversity across real-world settings, privacy-preserving data collection, and efficient deployment on edge and wearable hardware. In summary, this review consolidates recent progress in egocentric multi-view image analysis from 2024 to 2026 and identifies key research gaps that must be addressed to enable scalable, real-time, and socially responsible deployment. We argue that the next frontier lies in developing embodied, continually learning agents that seamlessly integrate multi-view perception, multimodal reasoning, and open-world adaptation in everyday environments.

## Figures and Tables

**Table 1:** Comparison of representative datasets.

Dataset	Year	Views	Scale	Modalities	Primary Tasks
Panoptic Studio [110]	2015	500+ cams	700 sequences	RGB, depth	3D pose, social interaction
InterHand2.6M [111]	2020	10+ cams	2.6M frames	RGB	3D hand pose
H_2_O [112]	2021	Multi-view	100K frames	RGB-D	HOI
HOT3D [6]	2025	Multi-view	833 min	RGB, IR, depth	3D hand–object tracking
EPIC-KITCHENS-100 [113]	2022	Ego	100+ h	RGB, audio	Action recognition
Ego4D [4]	2022	Ego	3000+ h	RGB, audio, IMU	Recognition, anticipation
Ego–Exo4D [5]	2024	Ego + Exo	100+ h	RGB, audio	Cross-view understanding
MultiEgo [19]	2025	Ego + Exo	Multi-session	RGB, IMU	4D reconstruction

**Table 2:** 3D hand pose estimation and reconstruction models on the InterHand2.6M [111] Dataset.

Model	View	Architecture	MPJPE (mm) ↓	MPVPE (mm) ↓	Accel. (mm/s^2^) ↓
Ren et al. [119]	Single	Decoupled Iterative Refinement	10.49	10.26	6.28
Pavlakos et al. [120]	Single	Transformers	9.84	10.13	5.13
Pan et al. [121]	Single	Feature Fusion	9.38	9.61	—
Moon et al. [122]	Single	Conditional Hand Modulator	—	9.40	—
Ren et al. [117]	Single (Video)	Temporal Convolution	7.21	7.39	4.54
Yu et al. [118]	Single (Video)	Generative Infilling + SLAM	7.94	8.15	**2.76**
Feng et al. [115]	Multi	Extract-Regress Network	6.65	7.00	—
Han et al. [116]	Multi	Cooperative Attention	**5.65**	**5.87**	—

Note: **Bold** indicates best results. ↓ indicates lower is better. — indicates metric not reported or not applicable. (e.g., Frame-based methods lack Accel. metrics; joint-only methods lack MPVPE).

**Table 3:** Single-view and multi-view hand-object interaction performance comparison on HOT3D [6].

Device	Single-View	Multi-View	Metric	Single	Multi	Gain
*3D hand tracking*						
Q3	UmeTrack [123]	UmeTrack-2V	MKPE ↓	18.0	**13.1**	27.2%
Q3	UmeTrack [123] + HOT3D [6]	UmeTrack + HOT3D-2V	MKPE ↓	15.4	**10.9**	29.2%
*6DoF object pose*						
Aria	FoundPose-1V [124]	FoundPose-3V	R@10 ↑	41.7	**52.9**	26.9%
Q3	FoundPose-1V [124]	FoundPose-2V	R@10 ↑	46.6	**55.9**	20.0%
*In-hand object lifting*						
Aria	MonoDepth	StereoMatch-3V, GT mask	R@10 ↑	30.2	**86.2**	185.4%
Aria	MonoDepth	StereoMatch-3V, pred. mask	R@10 ↑	23.3	**56.4**	142.1%
Q3	N/R	StereoMatch-2V, GT mask	R@10 ↑	–	**96.8**	–
Q3	N/R	StereoMatch-2V, pred. mask	R@10 ↑	–	**75.3**	–

*Note:* **Bold** indicates the best results. Q3 denotes Quest 3. MKPE is reported in mm. R@10 denotes recall at 10 cm for object lifting, and 10 cm/10° for 6DoF object pose. Gain is relative error reduction for MKPE and relative recall improvement for R@10. N/R means no single-view result was reported.

**Table 6:** Consolidated meta-analysis of multi-view versus single-view performance gains across all tasks and datasets surveyed in this review. Gain is relative error reduction for error metrics (↓) and relative or absolute improvement for accuracy/recall metrics (↑), as reported in the corresponding source table.

Task	Dataset	Metric	Reported Gain (Multi vs. Single)
3D hand pose & reconstruction	InterHand2.6M (Table 2)	MPJPE/MPVPE ↓	≈30%
3D hand tracking	HOT3D (Table 3)	MKPE ↓	27.2–29.2%
6DoF object pose	HOT3D (Table 3)	R@10 ↑	20.0–26.9%
In-hand object lifting	HOT3D (Table 3)	R@10 ↑	142.1–185.4%
Multimodal action segmentation	MultiEgo-style benchmarks (Section 4.3)	Segmentation metrics ↑	10–25%
Keystep recognition (naive fusion)	Ego–Exo4D (Table 5)	Accuracy ↑	−5.40% to +2.2%
Keystep recognition (view-aware fusion)	Ego–Exo4D (Table 5)	Accuracy ↑	+0.72 pt (52.36→53.08)
Proficiency estimation	Ego–Exo4D (Table 5)	Accuracy ↑	+0.7 to +4.25 pt

## Data Availability

No new data were created or analyzed in this study. Data sharing is not applicable to this article.
